# RETRACTION: Effects of Quality Nursing on the Surgical Site Wound Infection after Colostomy in Patients With Colorectal Cancer: A Meta‐Analysis

**DOI:** 10.1111/iwj.70277

**Published:** 2025-03-03

## Abstract

**RETRACTION**: LiuC. , HuangP. , HuH. , ChenG.‐W. , and ZhuL. , “Effects of Quality Nursing on the Surgical Site Wound Infection after Colostomy in Patients with Colorectal Cancer: A Meta‐Analysis,” International Wound Journal 21, no. 2 (2024): e14484, 10.1111/iwj.14484.PMC1082852142052948

The above article, published online on 30 January 2024, in Wiley Online Library (http://onlinelibrary.wiley.com/), has been retracted by agreement between the journal Editor in Chief, Professor Keith Harding; and John Wiley & Sons Ltd. Following an investigation by the publisher, all parties have concluded that this article was accepted solely on the basis of a compromised peer review process. The editors have therefore decided to retract the article. The authors did not respond to our notice regarding the retraction.

**RETRACTION**: C. Liu , P. Huang , H. Hu , G.‐W. Chen , and L. Zhu , “Effects of Quality Nursing on the Surgical Site Wound Infection after Colostomy in Patients with Colorectal Cancer: A Meta‐Analysis,” International Wound Journal 21, no. 2 (2024): e14484, 10.1111/iwj.14484.PMC1082852142052948

The above article, published online on 30 January 2024, in Wiley Online Library (http://onlinelibrary.wiley.com/), has been retracted by agreement between the journal Editor in Chief, Professor Keith Harding; and John Wiley & Sons Ltd. Following an investigation by the publisher, all parties have concluded that this article was accepted solely on the basis of a compromised peer review process. The editors have therefore decided to retract the article. The authors did not respond to our notice regarding the retraction.

